# Highly Thermally Conductive Polymer/Graphene Composites with Rapid Room-Temperature Self-Healing Capacity

**DOI:** 10.1007/s40820-022-00882-w

**Published:** 2022-06-15

**Authors:** Huitao Yu, Can Chen, Jinxu Sun, Heng Zhang, Yiyu Feng, Mengmeng Qin, Wei Feng

**Affiliations:** 1 School of Materials Science and Engineering and Tianjin Key Laboratory of Composite and Functional Materials, Tianjin University, Tianjin, 300350 People’s Republic of China; 2 Key Laboratory of Materials Processing and Mold, Ministry of Education, Zhengzhou University, Zhengzhou, 450002 People’s Republic of China

**Keywords:** Carbon/polymer composites, Self-healing capacity, High thermal conductivity, Molecular simulation, Room temperature

## Abstract

Composites that can rapidly self-healing their structure and function at room temperature have broad application prospects. However, in view of the complexity of composite structure and composition, its self-heal is facing challenges. In this article, supramolecular effect is proposed to repair the multistage structure, mechanical and thermal properties of composite materials. A stiff and tough supramolecular frameworks of 2-[[(butylamino)carbonyl]oxy]ethyl ester (PBA)–polydimethylsiloxane (PDMS) were established using a chain extender with double amide bonds in a side chain to extend prepolymers through copolymerization. Then, by introducing the copolymer into a folded graphene film (FGf), a highly thermally conductive composite of PBA–PDMS/FGf with self-healing capacity was fabricated. The ratio of crosslinking and hydrogen bonding was optimized to ensure that PBA–PDMS could completely self-heal at room temperature in 10 min. Additionally, PBA–PDMS/FGf exhibits a high tensile strength of 2.23 ± 0.15 MPa at break and high thermal conductivity of 13 ± 0.2 W m^−1^ K^−1^; of which the self-healing efficiencies were 100% and 98.65% at room temperature for tensile strength and thermal conductivity, respectively. The excellent self-healing performance comes from the efficient supramolecular interaction between polymer molecules, as well as polymer molecule and graphene. This kind of thermal conductive self-healing composite has important application prospects in the heat dissipation field of next generation electronic devices in the future.

**Supplementary Information:**

The online version contains supplementary material available at 10.1007/s40820-022-00882-w.

## Highlights

- PBA–PDMS/folded graphene film (FGf) composite with high strength and thermal conductivity and rapid self-healing capacity at room temperature is prepared.
- PBAx–PDMS/FGf self-healed its strength, thermal conductivity, and conductivity and the self-healing mechanism of thermal conductivity is also proposed.
- PBA–PDMS/FGf has significant application potential in interface thermal conductivity and robot skins.

**Supplementary Information:**

The online version contains supplementary material available at 10.1007/s40820-022-00882-w.

## Graphical abstract

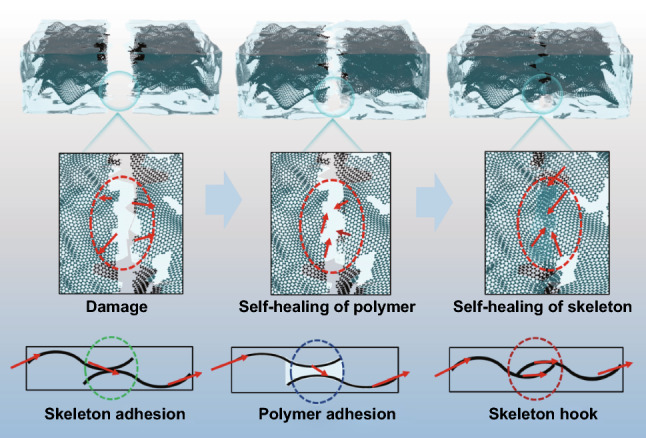

## Introduction

Polymer-matrix composites are valuable functional structural materials with several applications owing to their low weight, chemical resistance, and processability [1–3]. However, these materials exhibit degraded mechanical, thermal, and electrical properties in practical applications when subjected to irreversible mechanical damage during repeated deformation and friction. Self-healing materials can automatically repair any induced damage. In this context, if polymer-based composites that are subjected to considerable loading or operate in harsh environments are endowed with self-healing abilities, their durability and service life can be enhanced [4], which is significant for designing next-generation thermal conductive composites.

An effective strategy for designing self-healing polymer thermal conductive composites is the introduction of a self-healing polymer matrix in fillers. The self-healing ability of a polymer is mainly induced by the reversible interaction of molecules, reconstruction of chemical bonds, and movement or dynamic exchange of chains [5–7]. However, the mechanical strength of a material and its self-healing ability are mutually exclusive, i.e., materials with a high mechanical strength typically exhibit a limited chain mobility and high glass transition temperatures (*T*_g_), which limit their self-healing process. The realization of self-healing in materials necessitates demanding conditions such as high temperatures and humidity levels [8, 9]. Thus, the design and fabrication of polymers or their composites with rapid self-healing and excellent mechanical properties are challenging.

Optimization of the molecular crosslinking and dynamic reversible supramolecular interaction (π–π stacking, H-bonds, and metal–ligand) is a popular strategy for designing self-healing polymers [1, 10–13]. Specifically, through supramolecular interaction, damaged polymer segments can undergo molecular segment reconstruction and structural self-healing under different chemical stimulations or environments. Segments with dynamic reversible supramolecular interactions can help endow polymers, such as polysiloxane, polyether, and polyester, with self-healing capacities [14]. Moreover, the self-healing performance and mechanical strength of polymers can be enhanced by complementing reversible interactions with strong crosslinking, decreasing the *T*_*g*_ and selectivity of the self-healing reaction of the polymer, and promoting the movement of molecular segments. To this end, H-bonding is widely employed because of the associated directionality, affinity, fast-healing efficiency, adaptability, and simple self-healing conditions [15–18]. The organization of H-bonds to complement reversible interactions with strong crosslinking is promising for designing novel polymers with high strengths, suitable repair environments, and rapid self-healing capacities.

Graphene exhibits a high strength, thermal conductivity, and conductivity, and graphene-based fillers are widely used for reinforcing polymers to prepare functional composites for use in several application, including heat, electricity, and bionics [19–21]. However, compared with that of pristine polymers, thermal conductive composites exhibit more complex self-healing mechanisms owing to the chemical and structural differences between polymer and graphene fillers. Rigid graphene fillers or templates typically exhibit an inferior self-healing ability owing to restricted chain movement in the polymer matrix [22]. Furthermore, to enhance the performance of such thermal conductive composites, a high filler content is incorporated, which results in an uneven agglomeration of the fillers within the matrix and renders it challenging to reconstruct the fillers to form a network in the matrix after damage. The unhealable interface between the filler and polymers results in a significant decrease in the mechanical strength, conductivity, and thermal conductivity [23]. Therefore, reducing the filler content and optimizing the structural design of fillers are key issues for preparing self-healing thermal conductive composites.

Considering these aspects, this study was aimed at fabricating several self-healing materials with different molar ratios of 2-[[(butylamino)carbonyl]oxy]ethyl ester (PBA) and polydimethylsiloxane, vinyl end-blocked (PDMS). The PDMS was used as the molecular crosslinking segment, and PBA with intermolecular H-bonds was used as the dynamic supramolecular interaction segment. Subsequently, a composite (PBAx–PDMS/ folded graphene film (FGf)) with high strength and thermal conductivity and rapid self-healing capacity at room temperature was obtained using PBAx–PDMS polymer as the matrix and FGf as the conductive layer or skeleton under high vacuum pressure. Notably, PBAx–PDMS helps to control the microstructures of the composites, reconstruct the carbon-based phonon or electronics transmission channel at the fracture interface of PBAx–PDMS/FGf, and enhance the self-healing capacity and electrical and thermal conductivities of the composite. The PBAx–PDMS/FGf, which is expected to self-heal its strength, thermal conductivity, and electrical conductivity, has broad application prospects in thermal interface conductors and piezoresistive sensors.

## Experimental Section

### Materials

PBA and PDMS were purchased from Seans Co., Ltd., and 2,2'-azobis(2-methylpropionitrile) (AIBN) and ethyl acetate were provided by Ailan (Shanghai) Chemical Technology Co., Ltd. Dimethylacetamide (DMAC), a super dry solvent, was purchased from Seans Co., Ltd. Deionized water was prepared in the laboratory.

### Material Preparation

#### Synthesis of PBAx–PDMS

PBA and PDMS in different molar ratios (*m*_BCOE:PDMS_ = 1:1, 1:2, 1:3, 3:1, and 2:1) were added to 20 mL of DMAC and stirred at room temperature (the default room temperature in this study corresponds to 25 ℃, unless stated otherwise) for 2 h. Subsequently, 0.1 mmol, 16.41 mg AIBN was added and allowed to react at 67 °C for 24 h in an Ar environment. The reaction product was precipitated in deionized water, and the white precipitate was dissolved in ethyl acetate to obtain a mixed solution. The mixed solution was poured into a polytetrafluoroethylene mold, dried at room temperature for 12 h, and then dried at 60 °C for 24 h to obtain the sample. The sample was characterized through proton nuclear magnetic resonance (NMR) using CDCl_3_ as solvent (Fig. S1) (^1^H NMR; 10.19 (d, 1H, NH), 2.38 (s, 1H, CH_2_), 2.61 (s, 1H, CH), 3.99 (d, 2H, CH_2_), 4.14 (s, 2H, CH_2_), 3.24 (m, 2H, CH_2_), 1.12 (s, 2H, CH_2_), 1.24 (s, 2H, CH_2_), and 0.71 (s, 3H, CH_3_) ppm).

#### Synthesis of FGf and PBAx–PDMS/FGf

Folded graphene films were prepared using the template heat-shrink method (Fig. S2a-b). The graphene film was attached to a heat-shrinkable sheet with a thickness of 0.3 mm that contained adhesive on one side, and the sheet was shrunk through uniform heating from the edges using a heat gun at 150 °C (shrinkage ratio 1:5). Subsequently, the material was immersed in anhydrous ethanol for 12 h. The material was separated from the template to obtain the FGf. The FGf was soaked in a PBAx–PDMS/ethyl-acetate solution, dried at room temperature for 12 h, and then vacuum dried for 24 h. This process was repeated until the FGf was completely filled to obtain the PBAx–PDMS/FGf composite. The mass proportion of the FGf (Δ*M*) to the total mass of the composites (*m)* was calculated using Eq. 1, where *m*_FGf_ is the mass of the FGf.

1 $$ \Delta M = \frac{{m_{{{\text{FGf}}}} }}{m} \times 100\% . $$

### Mechanical Strength Before and After Self-healing

The mechanical tensile properties of the PBAx–PDMS and PBAx–PDMS/FGf composites were tested using an electron universal testing machine (XQ-46L, Baiying, China). The dimensions of the samples were 35.0 × 12.8 × 0.25 mm^3^, and a tensile rate of 5 mm min^−1^ was employed. The samples were loaded (0–50% compression), unloaded, and immediately reloaded 100 times. The polymer or composite was cut along the vertical stretching direction to induce fracturing. The material was fractured in close contact and maintained in this state for 0–2 h at room temperature. The self-healing efficiency of the tensile strength of the polymer or composites (*η*_σ_) was determined using Eq. 2:

2 $$ \eta_{\sigma } (\% ) = \frac{{\sigma_{{{\text{healed}}}} }}{{\sigma_{{{\text{pristine}}}} }} \times 100\% , $$

where *σ* is the maximum tensile strength.

### Self-healing Performance for Electrical or Thermal Conductivity

Electrical responses were measured using a TH2830 LCR meter. The thermal transport properties and thermal diffusivities of the samples (Φ10 × 3 mm) were also measured by the heat flow method (Xiangtan Xiangke, DRL-2A, China). The self-healing of the conductivity was measured considering the recovery of the brightness of a circuit bulb. The thermal transport and diffusivity of the samples (Φ10 × 3 mm) were evaluated by measuring the transmission temperature of the sensor before and after self-healing. All the composites were tested for 10 min, 0.5 h, 1 h, and 2 h. The self-healing efficiency of the electrical and thermal conductivities of the polymer and composites were determined from the ratio of the performance before and after self-healing.

### Molecular Simulation

First, the Amorphous Cell modeling was used to build the polymer into a box with dimensions of 33.043513 × 33.043513 × 33.043513 Å. Forcite, force fields, and simulated annealing methods were then used separately to relax the polymer structure with no atomic overlap. The specific method is as follows: heat up to 300–500 K, run NVT for 0.5 ns, then cool down to 500–300 K, then run NVT for 0.5 ns. Finally, molecular dynamics simulations were performed, when running NVT for 500 ps. The electrostatic potential and van der Waals forces were calculated using Atom based. And the Forcite was applied to calculate the binding energy.

## Results and Discussion

### Preparation and Characterization of PBA–PDMS/FGf Elastomers

**Fig. 1:**
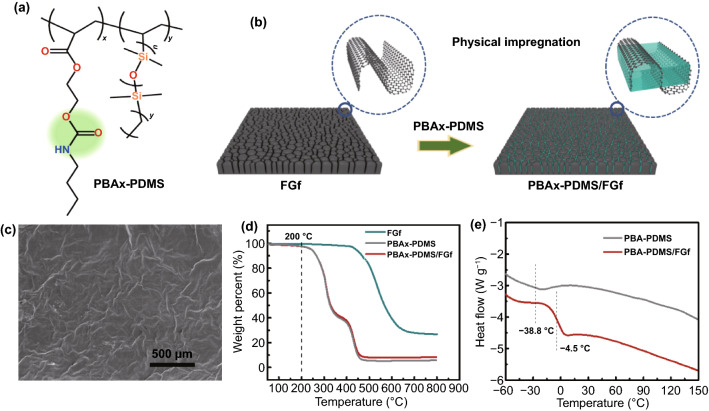
**a** Chemical structure of PBAx–PDMS. **b** Process of preparing PBAx–PDMS/FGf. **c** SEM images of PBAx–PDMS/FGf. **d** Thermogravimetric analysis (TGA) plots of PBAx–PDMS, FGf, and PBAx–PDMS/FGf. **e** Differential scanning calorimetry (DSC) curves of PBA–PDMS and PBA–PDMS/FGf

Several types of polymer materials with self-healing and high strength were designed and synthesized at varying proportions and distributions of different types of segments (soft and hard segment) in polymer chains [24]. In general, PBA is an acrylic polymer with double hydrogen bonds, and PDMS is a crosslinked polymer with double bonds at both ends. By controlling the ratio of PBA and PDMS, a series of self-healing polymer materials were prepared through random copolymerization (Fig. 1a). The PBAx–PDMS was synthesized through single-step radical polymerization from PBA monomers and PDMS, and the chemical structure of PBAx–PDMS was examined using the ^1^H NMR spectrum. The designed PBAx–PDMS/FGf elastomers were composed of self-healable PBA–PDMS and FGf, as shown in Fig. 1b. The PBAx–PDMS composite was filled into FGf through the dipping-pressure filling method (*m*_FGf_ = 2.17 wt%), and its micromorphology was characterized, as shown in Fig. 1c. Owing to the surface protrusions of the folded graphene, the surface of the material exhibited a continuous network distribution after the polymer was evenly filled into the gaps of the FGf (Fig. S2b). The continuous structure of graphene can enhance the mechanical properties of the composites, thereby providing a foundation for the popularization and application of the materials in thermal, electrical, and other aspects (Figs. S2c-d and S3).

In addition, the TGA results of FGF, PBAx–PDMS, and PBAx–PDMS/FGf showed that the first weight loss temperature of PBAx–PDMS/FGF composite was 200 °C (Fig. 1d). The higher weight loss temperature ensures the application of the material in thermal conductivity. Meanwhile, chain movement at a temperature is related to *T*_g_ of polymers with different molecular crosslinking configurations. In Fig. 1e, the *T*_g_ values of PBAx–PDMS and PBAx–PDMS/FGf elastomers were − 38.8 and − 4.5 °C, respectively. Compared with PBAx–PDMS, the *T*_g_ of PBAx–PDMS/ FGf was higher, which can be attributed to the presence of rigid FGf or templates that limit the movement and aggregate formation in the matrix. Notably, for PBAx–PDMS and PBAx–PDMS/FGf, the value of *T*_g_ < 0 °C, indicating that the reversible interaction of the material and the chain motion on the interface were high, thus ensuring that the polymer and its composites have the potential of rapid self-healing at room temperature [5, 20].

Moreover, the characteristic chemical structures of the PBA, PDMS, PBAx–PDMS, and PBAx–PDMS/FGf were investigated through Fourier transform infrared analyses (Fig. S4). The PBA exhibited stretching vibration peaks of C=O and C–N at 1725 and 1534 cm^−1^, respectively. The PDMS exhibited stretching vibration peaks of –Si–O– and C–N at 1065 and 1537 cm^−1^, respectively. The PBAx–PDMS exhibited stretching vibration peaks of N–H and –C–Si– at 3340 and 840 cm^−1^, respectively. The PBAx–PDMS/FGf exhibited peaks common to PBAx–PDMS, PBA, and PDMS. In the C 1*s* and N 1*s* X-ray photoelectron spectra of PBAx–PDMS (Fig. S5), peaks were observed at 284.06 and 398.18 eV, corresponding to C–N and –NH–. These results demonstrate that the integrity of the soft segment material was ensured and the hydrogen bonds of the material were retained.

### Molecular Simulation and Self-healing Properties of PBAx–PDMS

**Fig. 2:**
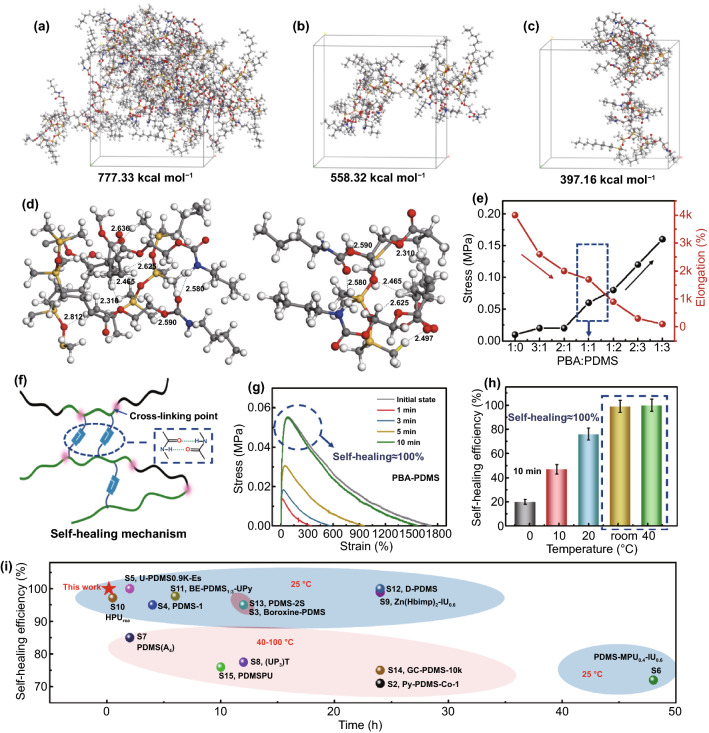
**a** Single point energy model for PBAx–PDMS. **b, c** Model for two chains with more forces in PBAx–PDMS. **d** Hydrogen bonding interactions between copolymers. **e** Strength and elongation of PBAx–PDMS for different molar ratios of PBA and PDMS. **f** Self-healing of polymer segments. **g** Stress–strain curves of the PBA–PDMS at room temperature for different self-healing durations (1, 3, 5, and 10 min). **h** Self-healing efficiency of PBA–PDMS at different temperatures (0, 10, 20, 25, and 40 °C) for 10 min. **i** Comparison of the self-healing efficiencies of the reported polymer with PDMS

Intermolecular hydrogen bonding can promote the rearrangement of polymer chain segments to induce the self-healing of the structure and properties of polymeric materials [25]. To verify the self-healing properties of PBAx–PDMS, the interfacial adhesion energy (IAE) of the prepared polymer was simulated in molecular simulations. In general, a higher IAE of polymerization corresponds to a more intense interfacial interaction of the material and superior self-healing properties. The molecular chains of PBA and PDMS with significant interactions were selected and modeled using an amorphous cell. The model of PBAx–PDMS was derived considering atomic no-recombination (Fig. 2a). Using the same principle, the model of the two chains was selected with more interaction forces on the PBAx–PDMS, and the results of model are shown in Fig. 2b, c. The maximum IAE of the PBAx–PDMS was 777.33 kcal mol^−1^, and the IAE of other models with more interaction forces were 558.32 and 394.36 kcal mol^−1^, respectively. According to the molecular simulations, the chain of PBA exhibited a satisfactory self-healing effect, and the strong interfacial adhesion of PBAx–PDMS could be achieved by adjusting the ratio of PBA to PDMS. In addition, hydrogen bonding interactions were observed on copolymers with the same or different monomer units in the same chain or between O (N) and H of different monomers in different chains (Fig. 2d). This result indicated the presence of a strong interfacial interaction between the polymer and graphene, and between the polymer and polymer, which lay a foundation for the preparation and application of subsequent composites.

The following three conditions must typically be satisfied to ensure satisfactory self-healing of the original polymer network at the macroscopically damaged interface: (i) interface contact, (ii) polymer–polymer or polymer–filler interactions, and (iii) effective load transfer at the interface. Therefore, the balance between strong crosslinking and rapid free chain movement is key to prepare robust PBAx–PDMS with rapid self-healing properties. To enhance the properties of the prepared polymer materials, the composition of the materials was optimized. Figure 2e shows the tensile properties of PBAx–PDMS (*x* = 1:0, 3:1, 2:1, 1:1, 1:2, 2:3, and 1:3) at room temperature. PBA_1:1_–PDMS (simplified as PBA–PDMS) exhibited a high tensile strength (0.06 ± 0.01 MPa) and elongation (1800%). The tensile strength of PBA–PDMS was lower than those of PBAx–PDMS (*x* = 1:2, 2:3, and 1:3). The elongation of PBA–PDMS was higher than those of PBA_x_–PDMS (*x* = 1:0, 3:1, 2:1). These findings indicated that the crosslinking using PDMS enhanced the strength of the polymer structure compared with that achieved using the H-bonding-linked structure of PBA, and the elongation of PBA led to the considerable elongation of the material structure. This aspect was also demonstrated by the highest elongation of PBA_1:0_–PDMS (4000%) at break among all considered materials. Therefore, PBA–PDMS, which combines the intermolecular H-bonds of PBA and crosslinking of PDMS, is an ideal candidate for high strength and elongation polymers. The PBA chain segments containing double hydrogen bonds can promote reversible interactions between polymeric linkages, facilitating the construction and self-assembly of chain segments. Therefore, PBA endows PBA–PDMS with self-healing functionalities.

In general, shorter molecular chain segments are more susceptible to intermolecular hydrogen bonding interactions (Fig. 2f). Therefore, PBAx–PDMS can achieve molecular reorganization and exhibit superior self-healing properties. The self-assembly of H-bonds depends on the healing temperature or time. The self-healing performance can be enhanced by the temperature-enhanced activity of H-bonds and segment mobility. The self-healing ability of PBA–PDMS at different healing durations or temperatures was examined. Figure 2g shows the self-healing performance of PBA–PDMS at room temperature. The self-healing efficiency (*η*_σ_) of the material increased as the healing time increased, and *η*_σ_ of PBA–PDMS reached 100% when the healing time was 10 min at room temperature. This phenomenon was attributable to the combined effect of the reversible interaction between neighboring H-bonds. The variations in the self-healing performance with the temperature and time were consistent with the previous analyses. The PBA–PDMS exhibited increasing self-healing efficiencies as the temperature increased, and the restoration efficiency was identical at room temperature and 40 °C (Fig. 2h). The PBA–PDMS exhibited an increased *η*_σ_ (100 ± 0.47%) at room temperature. This finding indicated that the optimal condition for the rapid self-healing of PBA–PDMS is room temperature for 10 min. In addition, PBA–PDMS exhibits a higher self-healing efficiency and lower self-healing temperature compared to the existing self-healing PDMS materials (Fig. 2i, Table S1). The material can rapidly recognize damage and self-heal at room temperature and is thus a promising candidate for operation at room temperatures.

### Self-healing Performance of PBA–PDMS/FGf

**Fig. 3:**
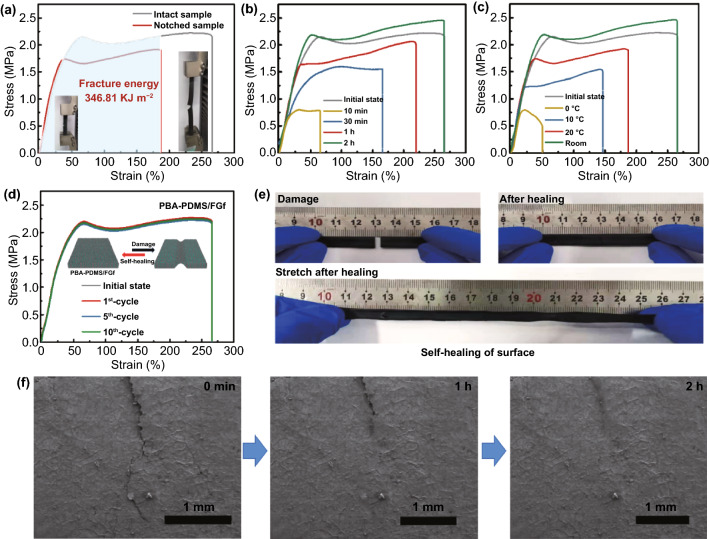
**a** Stress–strain curves for structurally complete and single-sided notched PBA–PDMS/FGf elastomers. The inset images show the elastomer at 180% strain (left) and 270% strain (right). **b** Stress–strain curves of the PBA–PDMS/FGf at room temperature for different healing durations (10 min, 30 min, 1 h, and 2 h). **c** Stress–strain curves of PBA–PDMS/FGf at different temperatures (0, 10, 20, and 25 °C) for 2 h. **d** Stress–strain curves of PBA–PDMS/FGf with 10 self-healing cycles at room temperature for 2 h. **e** Healing digital photographs and elasticity demonstration of the fractured PBA–PDMS/FGf elastomer. **f** Surface morphology images of the healed PBA–PDMS/FGf samples at room temperature for 0–2 h

For PBA–PDMS/FGf elastomers, the properties of composites deteriorated after irreversible damage or fracture when bending or interfacial stress concentration occurred in real-life applications. And the self-healing properties of composites are also influenced by the chemical activity and chain movement of the polymer segments [29]. Compared with the self-healing of electrical and thermal conductivities, the self-healing of mechanical properties is more challenging [26–28]. The elongation of FGf was 320% (Fig. S6a), and the tensile strength and elongation of PBA–PDMS/FGf were 2.23 ± 0.15 MPa and 270%, respectively, after the polymer was filled (Fig. S6b). As shown in Fig. S7, the width and thickness of the PBA–PDMS/FGf elastomer sheet were 10.0 and 3 mm, respectively. As shown in Fig. 3a, the tensile strength of PBA–PDMS/FGf with or without a single-sided notch (gap length of 1.0 mm) was tested at a rate of 5 mm min^−1^. The single-sided notched elastomer exhibited a maximum elongation of 180%. The fracture energy of PBA–PDMS/FGf was 346.81 ± 3.0 kJ m^−2^, determined using the Greensmith method [30]. Thus, the interactions of the PBA–PDMS are of significance in enhancing the damage tolerance of PBA–PDMS/FGf elastomers, and the material exhibits strong interfacial adhesion.

To quantitatively evaluate the self-healing ability of PBA–PDMS/FGf, the sheet was cut into two pieces and healed at room temperature for 0–2 h. As shown in Fig. 3b, the tensile strength of the PBA–PDMS/FGf film recovered to 0.75 ± 0.1 MPa, and *η*_σ_ was 34.09% after self-healing for 10 min. The film that self-healed for 1 h could be stretched to over 170% and exhibited a tensile strength of 1.5 ± 0.2 MPa with *η*_σ_ of 68.18%. When the self-healing time was 2 h, the healing efficiency of PBA–PDMS/FGf was similar to that in the initial state, and the mechanical properties were effectively healed. Moreover, the *η*_σ_ values of PBA–PDMS/FGf were 30.5 ± 1.50%, 56.81 ± 2.2%, 79.52 ± 2.50%, and 100% when the healing temperatures were 0, 10, 20, and 25 °C, respectively (Fig. 3c). As the self-healing duration increased, the properties of the material returned to the initial state, indicating that the chain movement of the polymers balanced the strong crosslinking and free chain movement, and ensured the structural integrity of the filler. Free-moving polymer chain segments typically enhance the self-healing efficiency of materials [31]. Moreover, because of the side-chain dual H-bonds, the PBA–PDMS/FGf could effectively eliminate damage. The mechanical properties of PBA–PDMS/FGf remained stable after 10 self-healing cycles at room temperature, indicating the stability of the self-healing function (Fig. 3d).

Figure 3e shows an image of the material damage, healing effect, and tensile strength after healing. The tensile strength of the material was restored after healing, and the structure was not fragmented. To verify the self-healing effect of PBA–PDMS/FGf, the morphology of the damage during the self-healing process was visually characterized using field-emission SEM (FESEM) (Fig. 3f). The damage of the PBA–PDMS/FGf, which was wide and deep before self-healing, appeared narrow and shallow after 1 h of self-healing. When the self-healing time was increased to 2 h, the damage completely disappeared, indicating that the material morphology could be completely healed with the extension of time. Compared to other polymeric carbon matrix composites, the composite has superior mechanical self-healing properties (low self-healing temperature, short self-healing time, Fig. S7). In order to verify the effect of self-healing of the structure on the self-healing of the thermal conductivity, the thermal conductivity self-healing properties were subsequently explored.

### Thermal Conductivity and Self-healing Efficiency for the PBA–PDMS/FGf

The main factors affecting the thermal conductivity of PBA-PDMS/FGf include: density, interfacial thermal resistance, and thermal conductivity channel. (i) In terms of density, the FGf materials have large voids between them on the macroscopic scale, and the density is low, so the thermal resistance is high. When filled with polymer, the density of the composite increases, resulting in higher thermal conductivity. (ii) When the ratio of PBA to PDMS is 1:1, PBA – PDMS and FGF have strong interface adhesion. The strong interfacial adhesion makes the composites have low interfacial thermal resistance. (iii) Hydrogen bonds have sufficient design flexibility and directionality to contribute significantly to the thermal conduction of molecular chains. Double hydrogen bonds can be formed between PBA molecules to improve the thermal connection between polymer chain segments. In contrast, PDMS can increase the degree of cross-linking of the polymer, allowing the polymer to take advantage of the rigidity of PDMS to promote an extended conformation with strong hydrogen bonds. Therefore, adjusting the ratio of PBA/PDMS in the polymer seriously affects the thermal conductivity of the composite.

**Fig. 4:**
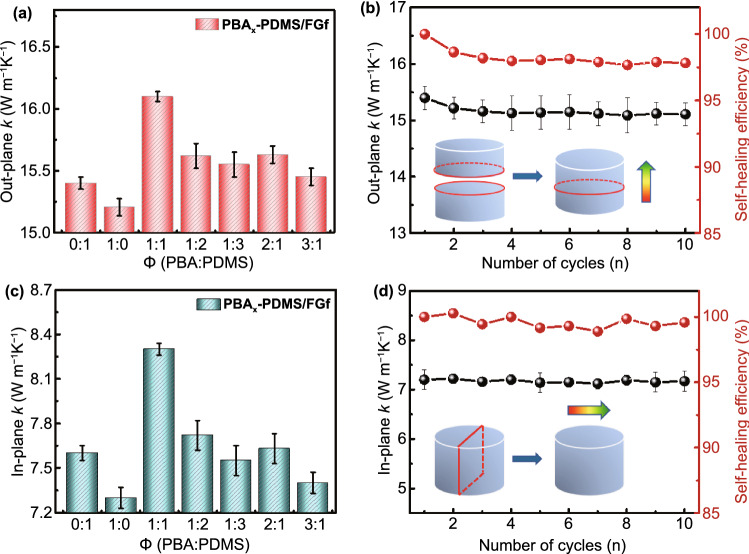
**a** Out-plane thermal conductivity and **b** self-healing efficiency of PBA–PDMS/FGf. **c** In-plane thermal conductivity and **d** self-healing efficiency of PBA–PDMS/FGf

The higher self-healing efficiency of the PBAx–PDMS/FGf composites was effective for maintaining a stable thermal conductivity. To examine the self-healing of thermal conductivity for PBA–PDMS/FGf composites, the out-plane and in-plane thermal conductivities of the materials before and after self-healing were evaluated. Figure 4a, b shows the out-plane thermal conductivity and self-healing efficiency of the PBA–PDMS/FGf after 10 self-healing cycles. With the increase in the ratio of PBA to PDMS, the thermal conductivity first increased and then decreased. When the ratio of PBA and PDMS was 1:1, the initial thermal conductivity of PBA–PDMS/FGf was the highest (13 ± 0.2 W m^−1^ K^−1^), and the self-healing efficiency after the first and tenth cycles were 98.65% and 97.83%, respectively. Moreover, the in-plane thermal conductivity and self-healing efficiency of the PBA–PDMS/FGf after 10 self-healing cycles were also evaluated (Fig. 4c, d). The thermal conductivity of in-plane and out-plane demonstrated the same variation trend. When the ratio of PBA to PDMS was 1:1, the initial thermal conductivity was 8.3 ± 0.2 W m^−1^ K^−1^, and the thermal conductivity self-healing efficiency was as high as 100% after 10 cycles. In summary, PBA–PDMS/FGf exhibited a high structural stability and could self-heal its thermal conductivity.

### Self-healing Mechanism for the Structure and Thermal Conductivity

**Fig. 5:**
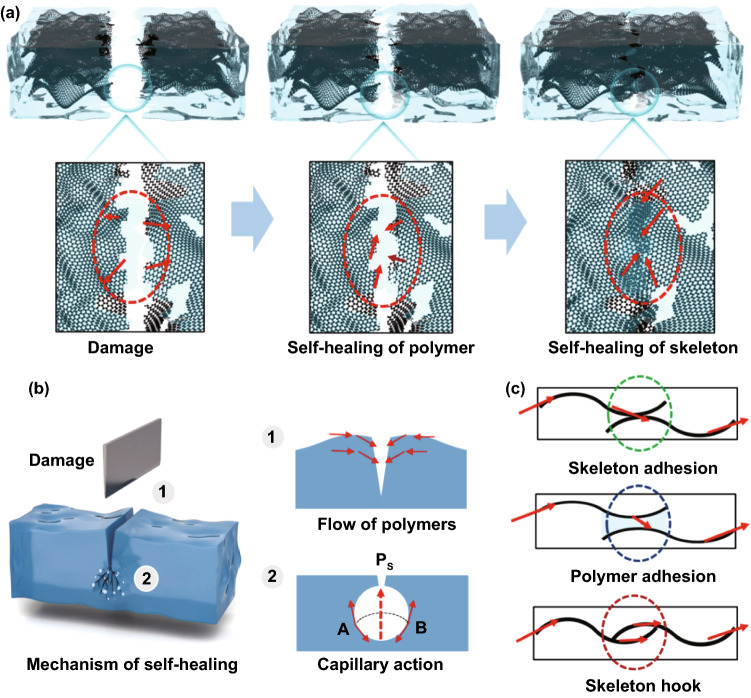
**a** Schematic of the self-healing process of PBA–PDMS/FGf. **b** Self-healing mechanism of macroscopic damage to materials. **c** Self-healing mechanism of PBA–PDMS/FGf for thermal conductivity

Unlike conventional polymer composites, the introduction of PBA–PDMS with excellent tensile properties and self-healing properties into the FGf allowed the PBA–PDMS/FGf composites material to spontaneously heal the damage or cracks, yielding a more stable structure and electrical and thermal functions. To explain the self-healing mechanism of the material structure and properties, Fig. 5a shows the schematic of the self-healing PBA–PDMS/FGf composite.

The PBA–PDMS/FGf composite was composed of a polymer matrix and the FGf filler. Therefore, the self-healing of the structure included polymerization and healing of the graphene framework. In terms of polymer molecules, the PBA–PDMS is a molecular segment with double hydrogen bonds. The directionality and rapid molecular rearrangement helped to eliminate the cracks, thereby achieving structural healing at room temperature. However, compared with the polymer matrix, the graphene filler was structurally stable, and the defects associated with fracturing prevented structural self-healing. In addition to the reversible interactions between the chain segment molecules of the polymer, some physical aspects of the interactions should also be considered (Fig. 5b). First, because of the influence of external force, it was easy to form bulges on both sides of the damage interface in the material structure. Therefore, the polymer in the composite flowed to the damage interface under the influence of gravity during self-healing, the contact effect of the material damage interface was improved, and the damage was self-healed. Second, the material contained a large number of bubbles in the interface holes at the damage, and the composite material flowed under the influence of capillary action to achieve the repair of macroscopic damage of the composite material [32, 33].

The effective thermal conductivity of the PBA–PDMS/FGf composite is influenced by FGf. Because of the higher IAE of PBA–PDMS materials, the self-healing of the thermal conductivity skeleton (path of thermal conductivity) can be divided into three types: (i) Adhering or bonding between the interface of FGfs; (ii) adhesion of polymer across the contact interface, resulting in the induction of capillary binding force and polymer chain entanglement; (iii) hooking between FGfs (Fig. 5c). These aspects allowed the polymer chain at the fracture interface to coordinate with the graphene framework for achieving rearrangement, proximity effect, and self-healing in thermal conductivity [34–38]. To verify the self-healing mode, the mechanical properties of the material in different states were used (Fig. S9). Three graphene membranes with the same morphology, structure and length were selected in the initial state. The mechanical properties of the composites after self-healing at different interfaces were compared with the mechanical strength of the initial state. The results showed that the self-healing efficiency of interfacial adhesion reached 76%, and the self-healing efficiency after polymer-linked fractured graphene was 35%. And these results indicate that the self-healing of the composite is mainly caused by the strong adhesion of the polymer to the graphene. And the data provide direct evidence of the primary mode of self-healing. Therefore, the composite material exhibited stable mechanical properties and thermal conductivity.

### Application of PBA–PDMS/FGf Elastomers

**Fig. 6:**
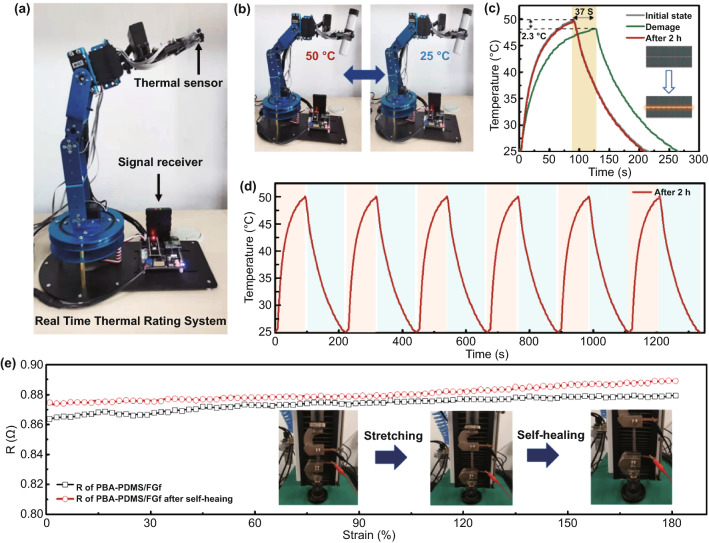
**a** Manipulator with sensor for temperature detection. **b** Digital images of the manipulator grabbing hot and cold bottles. **c** Temperature response of the composite material before and after the manipulator self-healed and grabbed the hot and cold bottles alternately. **d** Temperature response curves of PBA–PDMS/FGf that underwent six self-healing cycles at room temperature for 2 h. **e** Resistance of PBA–PDMS/FGf before and after self-healing in the stretched state

PBA–PDMS/FGf exhibits prompt self-healing capabilities, high thermal conductivity, high elasticity, and strong interfacial adhesion. Therefore, this material can be applied to prepare the artificial skin of an intelligent manipulator that can sense temperature changes through a temperature sensor. Compared with mechanical and electrical properties, the recovery of thermal conductivity by self-healing polymer/carbon composites is challenging owing to the difficultly in reconstructing the phonon migration path at the fracture interface of composites. Figure 6a shows the structural assembly of the manipulator. The temperature sensor is installed on the grab. When the temperature changes, the temperature sensing signal is transmitted to the computer.

Figure 6b shows an image of the manipulator grasping fragile glass bottles filled with cold water (25 °C) and hot water (50 °C). Because of the elasticity and interface adhesion of the material, when the manipulator grasped the hot bottle, the final temperature rapidly reached 50 °C (within 80 s). Subsequently, when the manipulator grasped the cold bottle, the sensor temperature decreased sharply, and the final temperature (25 ℃) was attained after 220 s. The difference in the actual and detected temperatures was minor, indicating the excellent heat transfer performance of the material. To evaluate the structural stability of PBA–PDMS/FGf, it was subjected to artificial in-plane separation (inset of Fig. 6c). When the manipulator grasped the hot bottle, the surface heating and cooling rates of the manipulator decreased (the heating and cooling durations were prolonged by 37 s), and the maximum temperature decreased by 2.3 °C, indicating that the thermal conductivity path of the material was damaged. When PBA–PDMS/FGf was allowed to self-heal at room temperature for 2 h, the thermal conductivity pathway was repaired, and the temperature profile returned to the initial state. To demonstrate the stability of the thermal conductivity self-healing of the material, the robot with self-healed PBA–PDMS/FGf alternately sensed the hot and cold bottles six times. The heating and cooling curves were stable, indicating the stability of the thermal conductivity of the material after self-healing (Fig. 6d).

The electrical resistance is a key parameter to measure the electrical conductivity of the PBA–PDMS/FGf material. Figure 6e shows the resistance of PBA–PDMS/FGf before and after self-healing in the stretched state. The resistance of PBA–PDMS/FGf was 0.865 ± 0.005 Ω in the tensile state. Subsequently, the PBA–PDMS/FGf sample was cut in the middle and place at room temperature for 2 h. The resistance after self-healing was 0.88 ± 0.005 Ω in the stretched state. The conductive channels of PBA–PDMS/FGf were self-healed at room temperature within 2 h, indicating that the material structure returned to its initial state after 2 h of self-healing, and dynamic re-healing of the structure could be realized at this resistance. Therefore, PBA–PDMS/FGf can be widely applied in the fields of electronics, aerospace and robot skins.

## Conclusions

In conclusion, optimized crosslinking and H-bonding network strategies, the polymer exhibited room-temperature self-healing capacities. It also received theoretical verification by the molecular simulation. When the ratio of PBA and PDMS is 1:1, the PBA–PDMS exhibits a high tensile strength (0.06 ± 0.01 MPa) and elongation (1800%). And the rapid self-healing condition of PBA–PDMS is room temperature for 10 min. In addition, a novel thermal conductive composite material with rapid room-temperature self-healing was prepared by physically impregnating PBA–PDMS copolymers in FGf. The initial out-plane and in-plane thermal conductivity of PBA–PDMS/FGf was 13 ± 0.2 and 8.3 ± 0.2 W m^−1^ K^−1^, respectively. The mechanical properties, and thermal conductivities of the PBA-PDMS/FGf were rapidly restored to their initial levels within 2 h after severing and autonomous healing. More importantly, it also explains the reconstruction of composite structure and heat conduction path in theory. On the whole, this research provides valuable reference for developing polymers and their composites with high strength and toughness, rapid stress recovery, wide temperature range, and self-healing capacities.

## Supplementary Information

Below is the link to the electronic supplementary material.

Supplementary file1 (PDF 503 KB)
