# Supplementary material for: Highly Thermally Conductive Polymer/Graphene Composites with Rapid Room-Temperature Self-Healing Capacity
Source: Nanomicro Lett. 2022 Jun 15;14:135. doi: 10.1007/s40820-022-00882-w (PMC9200911; doi:10.1007/s40820-022-00882-w)
Supplement: Supplementary file 1 — Supplementary file1 (PDF 503 KB) [file 40820_2022_882_MOESM1_ESM.pdf]

Supporting Information for

Highly Thermally Conductive Polymer/Graphene Composites with Rapid Room-Temperature Self-Healing Capacity

Huitao Yu[1], Can Chen[1], Jinxu Sun[1], Heng Zhang[1], Yiyu Feng[1, 2], Mengmeng Qin[1, *], Wei Feng[1, *]

[1] School of Materials Science and Engineering and Tianjin Key Laboratory of Composite and Functional Materials, Tianjin University, Tianjin 300350, P. R. China

[2] Key Laboratory of Materials Processing and Mold, Ministry of Education, Zhengzhou University, Zhengzhou 450002, P. R. China

*Corresponding authors. E-mail: qmm@tju.edu.cn (Mengmeng Qin), weifeng@tju.edu.cn (Wei Feng)

S1 Structural Analysis and Characterization

The morphology and microstructure of PBAx–PDMS and PBAx–PDMS/FGf composites were characterized by field-emission scanning electron microscopy (FESEM; S4800-15kV, Hitachi, Japan). The chemical structures and components of the copolymer samples were examined by Fourier transformation infrared spectroscopy (FT-IR; Tensor27, Bruker, Germany) and X-ray photoelectron spectroscopy (XPS; 250Xi, Thermo Fischer, USA), respectively. All C1s line was corrected at 284.6 eV. The crystallinity of the polymer and the phase of the composite were characterized by X-ray diffraction (XRD, D8 Advance, Bruker, Germany) with Cu Kα radiation (λ = 1.54 Å). The glass transition temperature (T_g) and of the samples was evaluated by differential scanning calorimetry (DSC; TA660, TA instrument, Japan) in an Ar atmosphere at heating and cooling rates of 10 °C min^{-1}. ^{1}H-NMR spectra were recorded using a 400-MHz spectrometer (NMR; AVANCE III, Bruker, Swiss). Specifically, the content of the copolymerization unit is tested using the internal standard method, and the signal for quantitative integration and the number of protons generating the signal are selected to be found based on the molecular weight of the monomer. In order to ensure the reproducibility of the experiment, the molar ratio of the feed is used for its description. The $CDCl_3$ (^{1}H at 7.26 ppm) was used as the solvent, and the splitting patterns of NMR were as follows: s, singlet; t, triplet; q, quartet; m, multiplet.

S2 Supplementary Figures and Tables

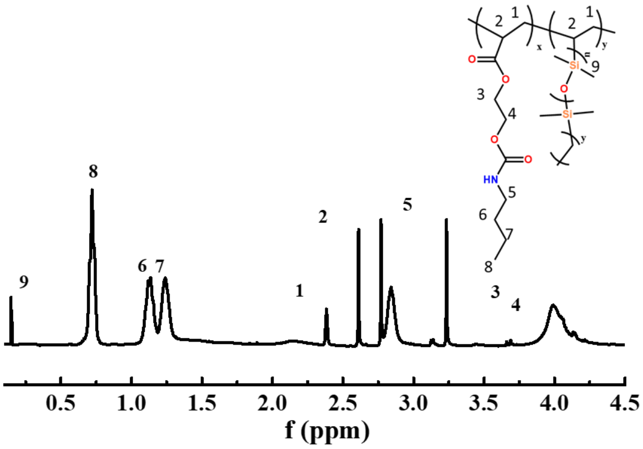

Fig. S1 ^{1}H NMR spectra of PBA-PDMS in $CDCl_3$

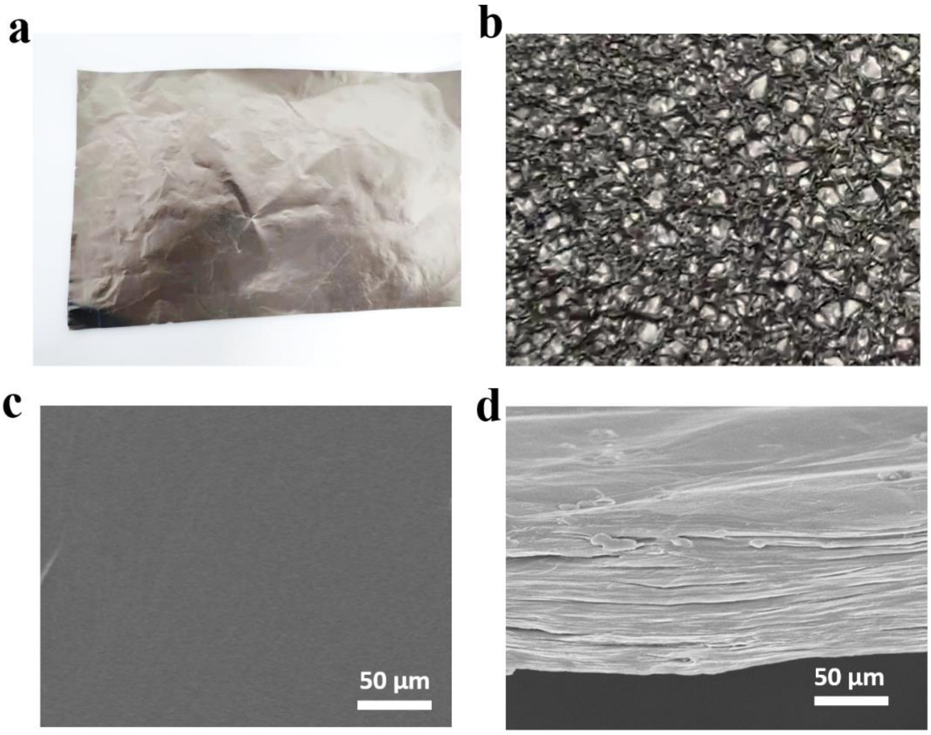

Fig. S2 (**a**) Digital electro-photograph of (**a**) graphene film and (**b**) FGf. The (**c**) surface and (**d**) cross sectional SEM image of FGf

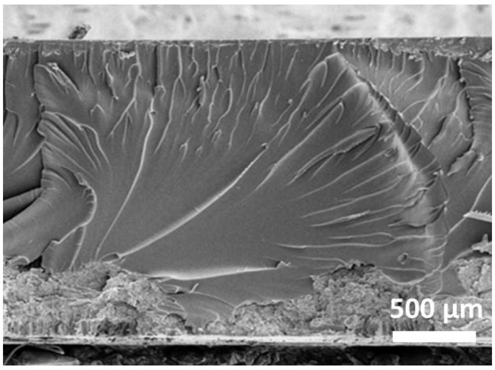

Fig. S3 Cross sectional SEM image of PBA-PDMS/FGf

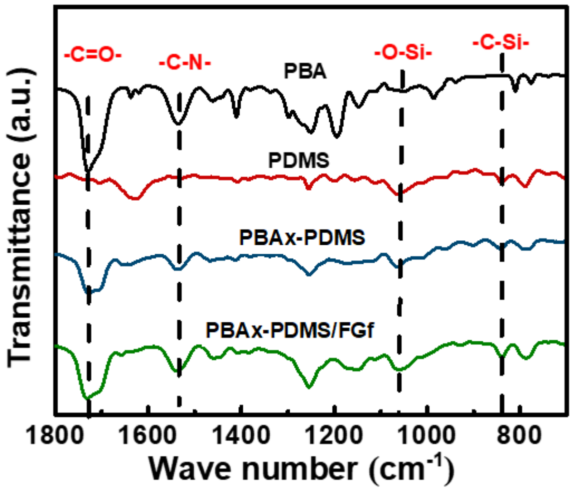

Fig. S4 FTIR spectra of PBA, PDMS, PBAx–PDMS, and PBAx–PDMS/FGf

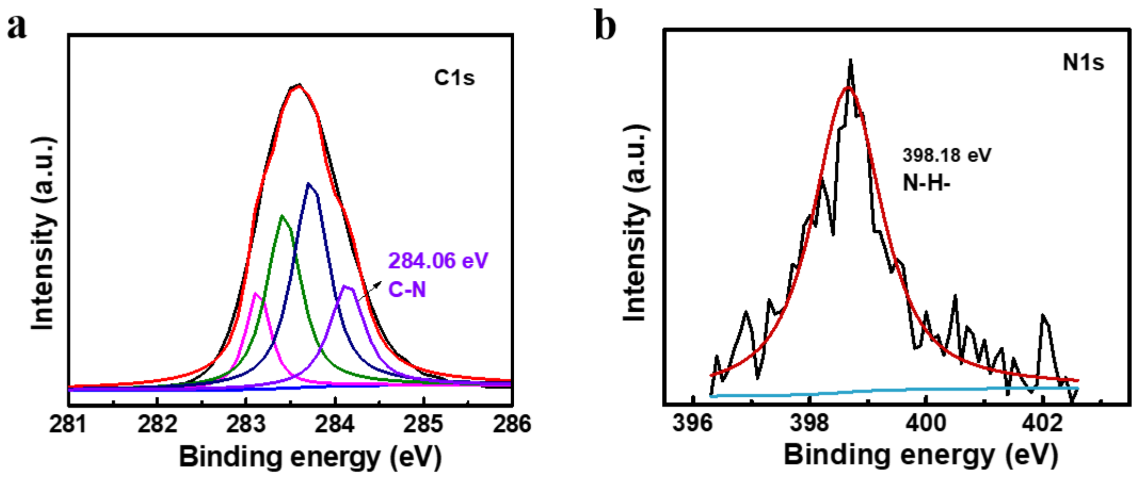

Fig. S5 Structural characterization of polymers. Magnification of (**a**) C1s, and (**b**) N1s of PBA-PDMS

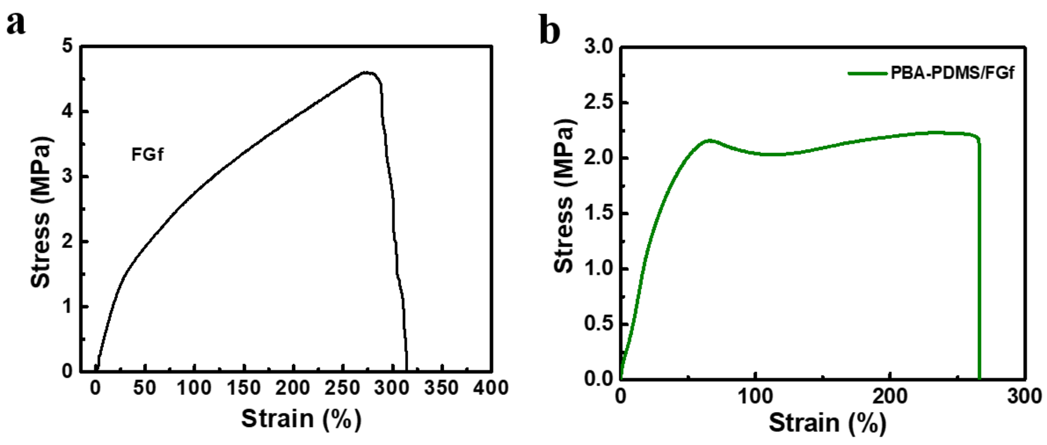

Fig. S6 (**a**) Stress-strain behavior of GFf and (**b**) PBA-PDMS/FGf at room temperature

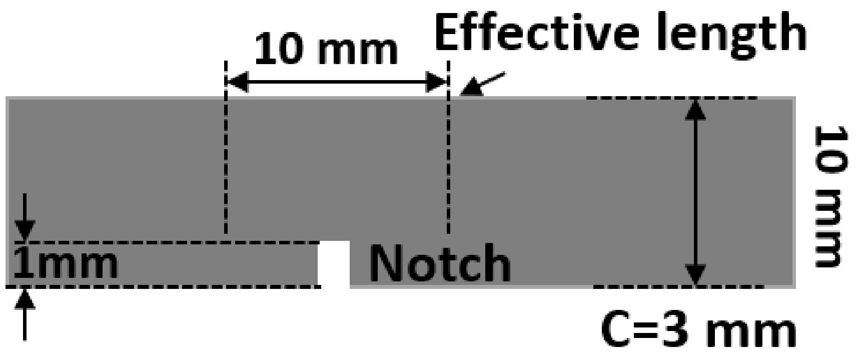

Fig. S7 Fracture energy test sample model

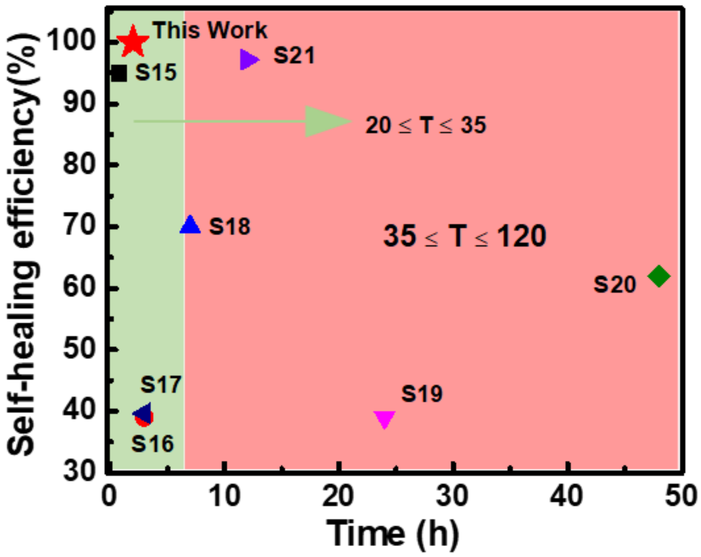

Fig. S8 Comparison of the self-healing efficiencies of the reported polymer compositions

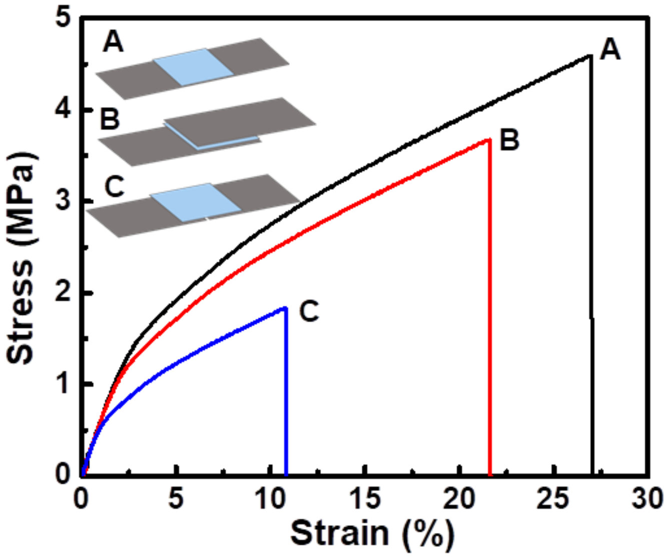

Fig. S9 Mechanical strength of composites with different composite methods

First, three graphene membranes with the same morphology, structure and length were selected in the initial state. In the initial state, the polymer composite is obtained by covering the graphene film with polymer, and its mechanical tensile curve is A. The same length of graphene is folded in half and filled with polymer in the middle, and its stretching curve is set to B. The graphene film breaks, and then the polymer is covered at the fracture, and its mechanical tensile curve is set as C. The results show that the highest mechanical strength of A in the intact state is 4.8 MPa. The mechanical strength of the composite in the B state is 3.65 MPa and the self-healing efficiency is 76%. The mechanical strength of the composite in the C state is 1.7 MPa and the self-healing efficiency is 35%. This indicates that the self-healing of the composite is mainly caused by the strong adhesion of the polymer to the graphene. And the data provide direct evidence of the primary mode of self-healing.

Table S1 Summary of the self-healing polymer materials reported in literatures

Sample	*E* (MPa)	*T* (ºC)	Time (h)	η_σ (%)	Refs.
Py-PDMS-Co-1	-	25	24	71	[S1]
Boroxine-PDMS	182 ± 15.8	70	12	95	[S2]
PDMS-1	2.34 ± 0.22	25	4	95	[S3]
U-PDMS0.9K-Es	0.23 ± 0.07	40	2	100	[S4]
PDMS–$MPU_{0.4}$–$IU_{0.6}$	0.62 ± 0.06	25	48	72	[S5]
PDMS (A_4)	-	100	2	85	[S6]
$(UP)_3T$	47.39 ± 1.03	40	12	77.5	[S7]
Zn $(Hbimcp)_2$-PDMS	43.68 ± 3.27	25	24	98.9 ± 1.9	[S8]
HPUrea	~ 0.12	25	0.5	97.2±2.2	[S9]
BE-$PDMS_{1:3}$-UPy	130.46±10	25	6	97.69±0.33	[S10]
D-PDMS	0.51	25	24	100	[S11]
PDMS-2S	0.32	25	12	95	[S12]
GC-PDMS-10k	0.17	80	24	75	[S13]
PDMSPU	0.0747	60	10	76	[S14]
PBA-PDMS	0.23±0.1	25	10 (min)	100	This work

The default room temperature is 25 °C.

Table S2 Summary of the graphene/polymers composites materials reported in literatures

Sample	σ (MPa)	*T* (ºC)	Time (h)	η_σ (%)	Refs.
graphene/PDMS–urea	17.5	50	0.8	95	[S15]
SHPU/grapheme	4	30	3	39	[S15]
NR/Graphene-2	4.59 ± 0.08	70	7	70	[S16]
PANDA/Gr-0.6	22.3 ± 1.9	75	24	39 ± 4	[S17]
PU-EDM/rmGO-1	31.16 ± 3.30	65	48	62	[S18]
PU/MG050	7.10	30	3	39.63	[S19]
AgNW/EVA	4	50	12	97.17	[S20]
This work	2.23 ± 0.15	25	2	100	

Supplementary References

[S1] J.C. Lai, J.F. Mei, X.Y. Jia, C.H. Li, X.Z. You et al., A stiff and healable polymer based on dynamic-covalent boroxine bonds. Adv. Mater. **28**(37), 8277-8282 (2016). https://doi.org/10.1002/adma.201602332

[S2] C. Lv, K. Zhao, J. Zheng, A highly stretchable self-healing poly (dimethylsiloxane) elastomer with reprocessability and degradability. Macromolecular Rap. Commun. **39**, 1700686 (2018). https://doi.org/10.1002/marc.201700686

[S3] M. Liu, P. Liu, G. Lu, Z. Xu, X. Yao, Multiphase-assembly of siloxane oligomers with improved mechanical strength and water-enhanced healing. Angew. Chem. **130**(35), 11412-11416 (2018). https://doi.org/10.1002/ange.201805206

[S4] P.F. Cao, B. Li, T. Hong, J. Townsend, Z. Qiang et al., Superstretchable, self-healing polymeric elastomers with tunable properties. Adv. Funct. Mater. **28**(22), 1800741 (2018). https://doi.org/10.1002/adfm.201800741

[S5] J. Kang, D. Son, G.J.N. Wang, Y. Liu, J. Lopez et al., Tough and water-insensitive self-healing elastomer for robust electronic skin. Adv. Mater. **30**(13), 1706846 (2018). https://doi.org/10.1002/adma.201706846

[S6] D.D. Zhang, Y.B. Ruan, B.Q. Zhang, X. Qiao, G. Deng et al., A self-healing PDMS elastomer based on acylhydrazone groups and the role of hydrogen bonds. Polymer **120**, 189-196 (2017). https://doi.org/10.1016/j.polymer.2017.05.060

[S7] M. Liu, P. Liu, G. Lu, Z. Xu, X. Yao, Multiphase-assembly of siloxane oligomers with improved mechanical strength and water-enhanced healing. Angew. Chem. **130**(35), 11412-11416 (2018). https://doi.org/10.1002/ange.201805206

[S8] J.C. Lai, X.Y. Jia, D.P. Wang, Y.B. Deng, P. Zheng et al., Thermodynamically stable whilst kinetically labile coordination bonds lead to strong and tough self-healing polymers. Nat. Commun. **10**, 1164 (2019). https://doi.org/10.1038/s41467-019-09130-z

[S9] D.P. Wang, Z.H. Zhao, C.H. Li, Universal self-healing poly (dimethylsiloxane) polymer crosslinked predominantly by physical entanglements. ACS Appl. Mater. Interfaces **13**(26), 31129-31139 (2021). https://doi.org/10.1021/acsami.1c06521

[S10] H. Yu, Y. Feng, L. Gao, C. Chen, Z. Zhang et al., Self-healing high strength and thermal conductivity of 3D graphene/PDMS composites by the optimization of multiple molecular interactions. Macromolecules **53**(16), 7161-7170 (2020). https://doi.org/10.1021/acs.macromol.9b02544

[S11] K. Zhang, J. Sun, J. Song, C. Gao, Z. Wang et al., Self-healing Ti_3C_2 MXene/PDMS supramolecular elastomers based on small biomolecules modification for wearable sensors. ACS Appl. Mater. Interfaces **12**(40), 45306-45314 (2020). https://doi.org/10.1021/acsami.0c13653

[S12] C. Lv, J. Wang, Z. Li, K. Zhao, J. Zheng, Degradable, reprocessable, self-healing PDMS/CNTs nanocomposite elastomers with high stretchability and toughness based on novel dual-dynamic covalent sacrificial system. Compos. Part B Eng. **177**, 107270 (2019). https://doi.org/10.1016/j.compositesb.2019.107270

[S13] L. Chen, H. Peng, Y. Wei, X. Wang, Y. Jin et al., Self-healing properties of PDMS elastomers via guanine and cytosine base pairs. Macromol. Chem. Phys. **220**, 1900280 (2019). https://doi.org/10.1002/macp.201900280

[S14] X. Zhao, J. Peng, S. Gao, K. Zhu, Y. Zhao et al., Self-healing anti-icing coatings prepared from PDMS polyurea. Sci. China Technol. Sci. **64**, 1535-1543 (2021). https://doi.org/10.1007/s11431-021-1831-7

[S15] M. Fang, D. Li, H. Lin, C. Luo, R. Qi et al., Flexible and recyclable conductive composite based on few-layered graphene with excellent self-healing capability. Europ. Polym. J. **108**, 536-541 (2018). https://doi.org/10.1016/j.eurpolymj.2018.09.005

[S16] M. Hernandez, M.M. Bernal, A.M. Grande, N. Zhong, S. Zwaag et al., Effect of graphene content on the restoration of mechanical, electrical and thermal functionalities of a self-healing natural rubber. Smart Mater. Struct. **26**(8), 085010 (2017). https://doi.org/10.1088/1361-665X/aa71f5

[S17] C. Cai, Y. Zhang, M. Li, Y. Chen, R. Zhang et al., Multiple-responsive shape memory polyacrylonitrile/graphene nanocomposites with rapid self-healing and recycling properties. RSC Adv. **8**(3), 1225-1231 (2018). https://doi.org/10.1039/C7RA11484B

[S18] C. Lin, D. Sheng, X. Liu, S. Xu, F. Ji et al., NIR induced self-healing electrical conductivity polyurethane/graphene nanocomposites based on diels− alder reaction. Polymer **140**, 150-157 (2018). https://doi.org/10.1016/j.polymer.2018.02.036

[S19] J.T. Kim, B.K. Kim, E.Y. Kim, S.H. Kwon, H.M. Jeong. Synthesis and properties of near IR induced self-healable polyurethane/graphene nanocomposites. Europ. Polym. J. **49**(12), 3889-3896 (2013). https://doi.org/10.1016/j.eurpolymj.2013.10.009

[S20] S. Liu, Z. Li, Q. Yu, Y. Qi, Z. Peng et al., Dual self-healing composite coating on magnesium alloys for corrosion protection. Chem. Eng. J. **424**, 130551 (2021). https://doi.org/10.1016/j.cej.2021.130551
